# The α9α10 Nicotinic Acetylcholine Receptor Antagonist αO-Conotoxin GeXIVA[1,2] Alleviates and Reverses Chemotherapy-Induced Neuropathic Pain

**DOI:** 10.3390/md17050265

**Published:** 2019-05-05

**Authors:** Huanbai Wang, Xiaodan Li, Dongting Zhangsun, Gang Yu, Ruibin Su, Sulan Luo

**Affiliations:** 1 Key Laboratory of Tropical Biological Resources, Ministry of Education, Key Laboratory for Marine Drugs of Haikou, Hainan University, Haikou, Hainan 570228, China; hbwang93@163.com (H.W.); lixiaodan816@163.com (X.L.); zhangsundt@163.com (D.Z.); 2 State Key Laboratory of Toxicology and Medical Countermeasures, Beijing Key Laboratory of Neuropsychopharmacology, Beijing Institute of Pharmacology and Toxicology, 27 Taiping Road, Beijing 100850, China

**Keywords:** αO-conotoxin GeXIVA[1,2], α9α10 nAChR, oxaliplatin-induced neuropathic pain, mechanical allodynia, cold allodynia

## Abstract

Oxaliplatin is a third-generation platinum drug and is widely used as a first-line therapy for the treatment of colorectal cancer (CRC). However, a large number of patients receiving oxaliplatin develop dose-limiting painful neuropathy. Here, we report that αO-conotoxin GeXIVA[1,2], a highly potent and selective antagonist of the α9α10 nicotinic acetylcholine receptor (nAChR) subtype, can relieve and reverse oxaliplatin-induced mechanical and cold allodynia after single and repeated intramuscular (IM) injections in rats. Treatments were started at 4 days post oxaliplatin injection when neuropathic pain emerged and continued for 8 and 16 days. Cold score and mechanical paw withdrawal threshold (PWT) were detected by the acetone test and von Frey test respectively. GeXIVA[1,2] significantly relieved mechanical and cold allodynia in oxaliplatin-treated rats after a single injection. After repeated treatments, GeXIVA[1,2] produced a cumulative analgesic effect without tolerance and promoted recovery from neuropathic pain. Moreover, the long lasting analgesic effect of GeXIVA[1,2] on mechanical allodynia continued until day 10 after the termination of the 16-day repeated treatment procedure. On the contrary, GeXIVA[1,2] did not affect acute mechanical and thermal pain behaviors in normal rats after repeated injections detected by the von Frey test and tail flick test. GeXIVA[1,2] had no influence on rat hind limb grip strength and body weight after repeated treatments. These results indicate that αO-conotoxin GeXIVA[1,2] could provide a novel strategy to treat chemotherapy-induced neuropathic pain.

## 1. Introduction

Cancer is a major threat to human health, and chemotherapy is one of the most effective treatments. However, a variety of chemotherapeutics including oxaliplatin can cause severe side effects during treatments, leading to painful symptoms that might result in the interruption of cancer treatment [1]. Unfortunately, few strategies are available for treating this kind of neuropathy, partially due to the complexity of its pathogenesis [2]. Therefore, it has great significance to develop analgesic drugs with novel mechanisms.

nAChRs are pentameric ligand-gated ion channels composed of different subunits (α1-α7, α9, α10, β1-β4, δ, ε, and γ) that can form various subtypes [3]. The diversity of nAChR subtypes enables them to regulate a variety of important physiological or pathological processes [4] including cancer [5], neurological disease [6], addiction [7], inflammation [8], pain [9], etc. The α9α10 nAChR is a newly discovered subtype, recently verified as a novel analgesic target to treat neuropathic pain [10,11]. Previous studies indicated the potency of multiple α9α10 nAChR antagonists in alleviating neuropathic pain in different kinds of rodent models, including trauma (chronic constriction injury, CCI) [12], disease (diabetes) [13], and inflammation (formalin) [14] induced neuropathic pain. Moreover, several of these antagonists produce a cumulative anti-nociceptive effect after repeated treatment that promotes recovery from neuropathy [15]. Recent studies on α-conotoxin RgIA, an antagonist of α9α10 nAChR, and its analogue RgIA4 verified α9α10 nAChR as a promising target for oxaliplatin-induced neuropathic pain treatment [16,17,18].

αO-conotoxin GeXIVA, a short peptide isolated from *conus generalis*, a kind of cone snail distributed in the South China Sea, was recently identified. This peptide is composed of 28 amino acids including four Cys residues that can form three different disulfide bond connection isomers, i.e., GeXIVA[1,2], GeXIVA[1,3], and GeXIVA[1,4], as previously described. Among them, GeXIVA[1,2] was the most potent antagonist of α9α10 nAChR (IC_50_ = 4.61 nmol at rat α9α10 nAChR) with a high specificity [19]. Previous studies have indicated the anti-hypersensitive effect of GeXIVA[1,2] in the rat CCI-induced neuropathic pain model. Repeated intramuscular (IM) injection of GeXIVA[1,2] produced a long-lasting cumulative analgesic effect that could continue for 2 weeks after termination of a 14-day daily treatment procedure [20].

The present study aimed to discover the possibility of applying GeXIVA[1,2] to control oxaliplatin-induced neuropathic pain. GeXIVA[1,2] was administered through single or repeated intramuscular (IM) injections in animals treated with oxaliplatin. Two common pain measurement methods, the von Frey test and the acetone test, were utilized to test the representative symptoms, mechanical and cold allodynia of this neuropathy. In normal rats, the influence of GeXIVA[1,2] on acute pain perception was detected by the von Frey test and tail flick test, and a grip strength test was performed to evaluate muscle strength following repeated treatments.

## 2. Results

### 2.1. Development of Neuropathic Pain in Oxaliplatin-Treated Rats

Rats receiving oxaliplatin developed neuropathic pain between 4–7 days post injection while the pain threshold of the glucose solution (GS) group did not change. The mechanical paw withdrawal threshold (PWT) decreased from 20.54 g ± 0.80 g (before) to 2.71 g ± 0.04 g (after). The cold score increased from 1.42 ± 0.19 (before) to 5.98 ± 0.15 (after). These results are similar to those obtained in previous research.

### 2.2. Analgesic Effects of GeXIVA[1,2] on Oxaliplatin-Induced Mechanical Allodynia 

#### 2.2.1. Analgesic Effect of GeXIVA[1,2] by Single Administration

**Figure 1:**
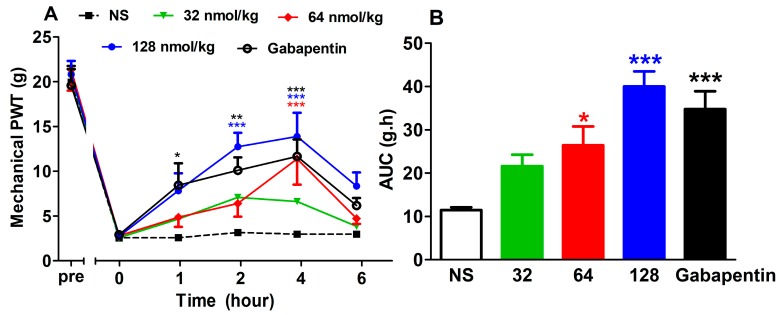
Analgesic effect of GeXIVA[1,2] on oxaliplatin-induced mechanical allodynia by single intramuscular (IM) injection. (**A**) Time–effect relationship of GeXIVA[1,2] on mechanical paw withdrawal threshold (PWT). Each point indicates the mean ± SEM at the time point, (NS, *n* = 13; GeXIVA[1,2], *n* = 13 (32 nmol/kg, 64 nmol/kg), *n* = 14 (128 nmol/kg); Gabapentin 50 mg/kg, *n* = 14). (**B**) Area under curve (AUC) of the corresponding dose within 6 h after injection indicating the dose–effect relationship. *P < 0.05, ^**^P < 0.01, ***P < 0.001, compared with the normal saline (NS) control group.

As shown in Figure 1, both GeXIVA[1,2] and gabapentin increased mechanical PWT after single injection (F(4,310) = 6.11, *P* = 0.0003). Gabapentin at 50 mg/kg produced analgesia from 1 h post injection and it lasted until 4 h post injection (*P* < 0.05). The mechanical PWT of the normal saline (NS) group did not change significantly during this whole period. A single IM injection of GeXIVA[1,2] produced dose-dependent analgesia on oxaliplatin-induced mechanical allodynia. Starting at 2 h post injection, GeXIVA[1,2] 128 nmol/kg increased mechanical PWT significantly and persisted to 4 h post injection (*P* < 0.001). The medium dose of GeXIVA[1,2] (64 nmol/kg) also significantly increased mechanical PWT at 4 h post injection (*P* < 0.001).

The area under curve (AUC) of different treatment groups was calculated including data at 0, 1, 2, 4, and 6 h post drug injection (Figure 1B) (F(4,62) = 11.06, *P* < 0.0001). The AUC of GeXIVA[1,2] at 128 nmol/kg significantly increased compared with the NS group (*P* < 0.001), which is comparable to that of the gabapentin 50 mg/kg group (*P* < 0.001). The minor dose of GeXIVA[1,2] at 64 nmol/kg also produced a significant increase in AUC (*P* < 0.05).

#### 2.2.2. Acute Analgesic Effect of GeXIVA[1,2] Following Repeated Injections

**Figure 2:**
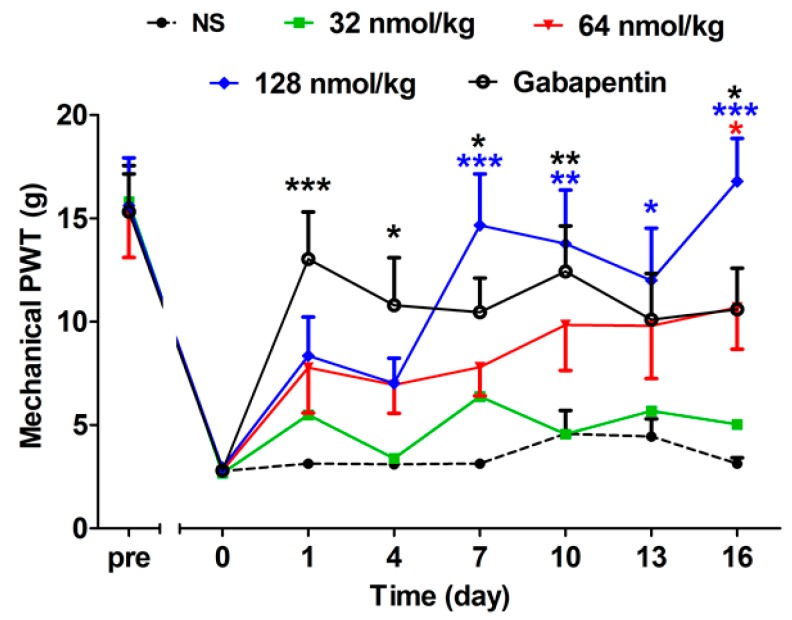
Acute analgesic effect of GeXIVA[1,2] on oxaliplatin-induced mechanical allodynia following repeated injections. Each data point indicates the mean ± SEM of mechanical PWT at 4 h post injection, (NS, *n* = 10; GeXIVA[1,2], *n* = 10 (32 nmol/kg, 64 nmol/kg, 128 nmol/kg), Gabapentin 50 mg/kg, *n* = 10). * *P* < 0.05, ** *P* < 0.01, *** *P* < 0.001, compared with the NS control group.

As indicated in Figure 1, both GeXIVA[1,2] and gabapentin increased mechanical PWT after single injection. Aiming to study the acute analgesic effect by repeated treatments, the mechanical PWTs of different drug treated rats were detected at 4 h post injection during a 16-day repeated treatment procedure (Figure 2) (F(4,315) = 10.07, *P* < 0.0001). The analgesic effect of GeXIVA[1,2] at 128 nmol/kg increased during the 16-day repeated treatment procedure and displayed a significant difference from the NS group between day 7 and day 16 (*P* < 0.05). The mechanical PWT of the GeXIVA[1,2] 64 nmol/kg-treated group displayed an upward trend and showed a significant difference at day 16 compared with the NS group (*P* < 0.05). The low dose of GeXIVA[1,2] at 32 nmol/kg showed little effect in mechanical PWT. Gabapentin at 50 mg/kg exhibited a stable analgesic effect at 4 h post treatment from day 1 and this lasted until the end of the trial except for day 13 (*P* < 0.05). The NS treatment had no effect on the paw withdrawal threshold at any time point during the experiment period.

#### 2.2.3. Short-Term Analgesic Effect of GeXIVA[1,2] by Repeated Treatments

**Figure 3:**
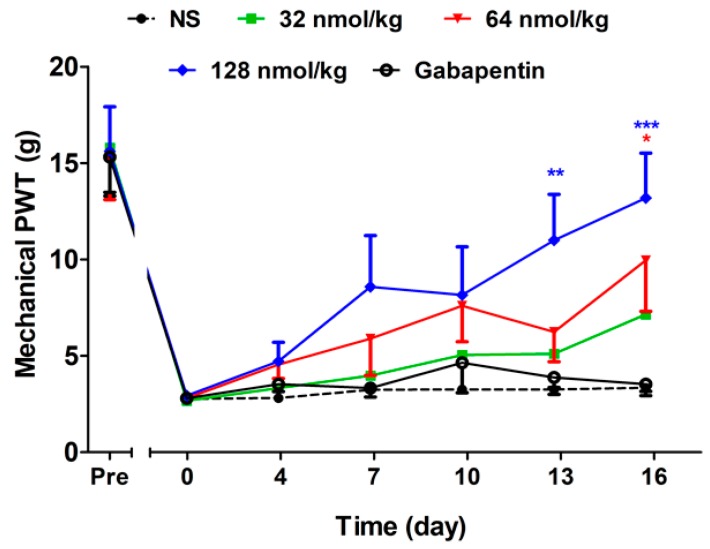
Short-term analgesic effect of GeXIVA[1,2] on oxaliplatin-induced mechanical allodynia by repeated treatments. Each data point represents the mean ± SEM of mechanical PWT at 24 h since last injection (NS, *n* = 10; GeXIVA[1,2], *n* = 10 (32 nmol/kg, 64 nmol/kg, 128 nmol/kg); Gabapentin 50 mg/kg, *n* = 10). * *P* < 0.05, ** *P* < 0.01, *** *P* < 0.001, compared with the NS control group.

As demonstrated in Figure 3, the short term analgesic effects of GeXIVA[1,2] and gabapentin at 24 h post injection were identified (F(4,270) = 4.12, *P* = 0.0063). Supportive evidence for the cumulative effects of GeXIVA[1,2] was found, indicated by the significant increase in mechanical PWT after repeated treatments. The mechanical PWT of the GeXIVA[1,2] 128 nmol/kg group exhibited an upward trend from day 4 and showed significant differences at day 13 and day 16 post drug injection compared with the NS group (*P* < 0.01). The lower dose of GeXIVA[1,2] at 64 nmol/kg exhibited a similar trend and showed a significant difference from the NS group at day 16 post drug injection (*P* < 0.05). On the contrary, gabapentin 50 mg/kg did not show analgesia at any time point. During the whole period, there was no significant difference in the mechanical PWT of the NS group (*P* > 0.05).

#### 2.2.4. Long-Term Analgesic Effect of GeXIVA[1,2] by Repeated Treatments

**Figure 4:**
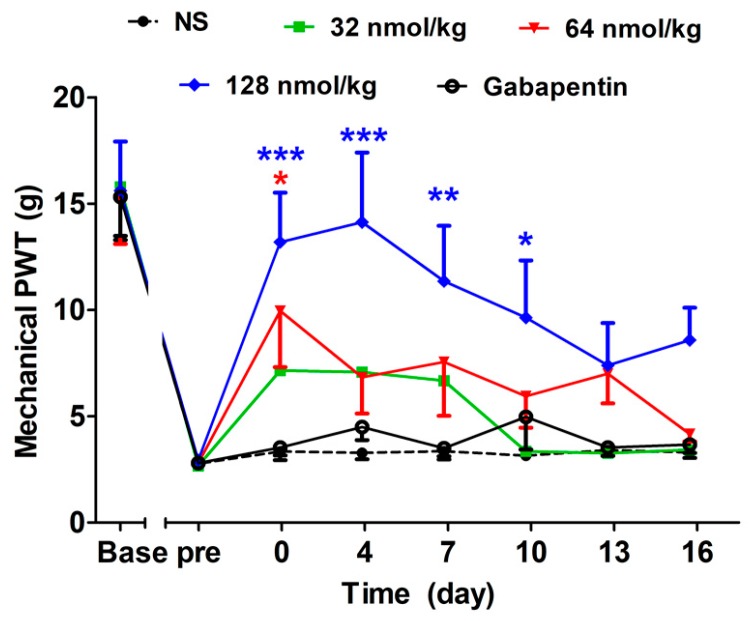
Long-term analgesic effect of GeXIVA[1,2] on oxaliplatin-induced mechanical allodynia. Mechanical PWT was observed after drug withdrawal from a 16-day repeated treatment. Each data point indicates the mean ± SEM of mechanical PWT at different time points, (NS, *n* = 10; GeXIVA[1,2], *n* = 10 (32 nmol/kg, 64 nmol/kg, 128 nmol/kg); Gabapentin 50 mg/kg, *n* = 10). * *P* < 0.05, ** *P* < 0.01, *** *P* < 0.001, compared with the NS control group.

For the purpose of studying the long-term analgesic effects of GeXIVA[1,2] and gabapentin after drug withdrawal from repeated treatments, the mechanical paw withdrawal thresholds of different treatment groups were continuously monitored for another 16 days after the 16 day treatment procedure was terminated. As described in Figure 4 (F(4,315) = 6.62, *P* = 0.0003), the GeXIVA[1,2] 128 nmol/kg group produced long-lasting analgesia after drug withdrawal, which lasted until day 10 after treatment termination (*P* < 0.05). After treatment finished, mechanical PWT for the lower dose of GeXIVA[1,2](64 nmol/kg) fluctuated and no significant difference was found compared to the NS group. The low dose of GeXIVA[1,2] (32 nmol/kg) or gabapentin at 50 mg/kg did not affect the mechanical PWT after drug withdrawal at any time point. There was no significant difference in the mechanical PWT of the NS group during the whole period.

### 2.3. Analgesic Fffects of GeXIVA[1,2] on Oxaliplatin-Induced Cold Allodynia

#### 2.3.1. Analgesic Effect of GeXIVA[1,2] by Single Administration

**Figure 5:**
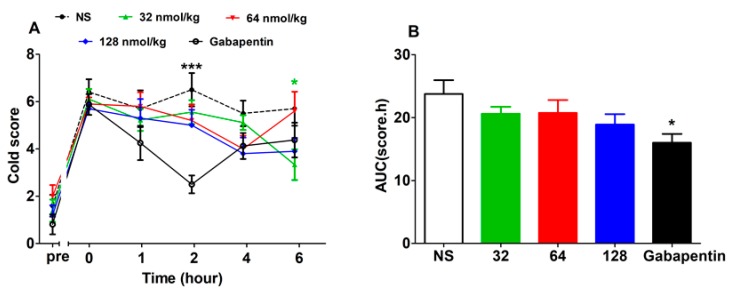
(**A**) Analgesic effect of GeXIVA[1,2] on oxaliplatin-induced cold allodynia by single IM injection. Time–effect relationship of GeXIVA[1,2] in acetone stimulation cold score. Each point indicates the mean ± SEM at the time point, (NS, *n* = 10; GeXIVA[1,2], *n* = 9 (32 nmol/kg), *n* = 10, (64 nmol/kg, 128 nmol/kg); Gabapentin 50 mg/kg, *n* = 8). (**B**) AUC of corresponding dose within 6 h after injection indicating the dose–effect relationship. * *P* < 0.05, *** *P* < 0.001, compared with the NS control group.

As described in Figure 5A (F (4,210) = 2.48), P = 0.0586), gabapentin at 50 mg/kg was able to attenuate oxaliplatin-induced cold allodynia at 2 h post drug injection (*P* < 0.001), while there was no significant change in the cold score of the NS group at any time point. On the contrary, GeXIVA[1,2] is inferior in alleviating cold allodynia, since only the 32 nmol/kg GeXIVA[1,2] group exhibited analgesia at 6 h post injection (*P* < 0.05). For the AUC (Figure 5B) (F (4,42) = 2.396, P = 0.0655), only the gabapentin 50 mg/kg group decreased compared with the NS group (*P* < 0.05); no significant difference between the NS group and all the GeXIVA[1,2] groups was observed.

#### 2.3.2. Short-Term Analgesic Effect of GeXIVA[1,2] by Repeated Treatments

**Figure 6:**
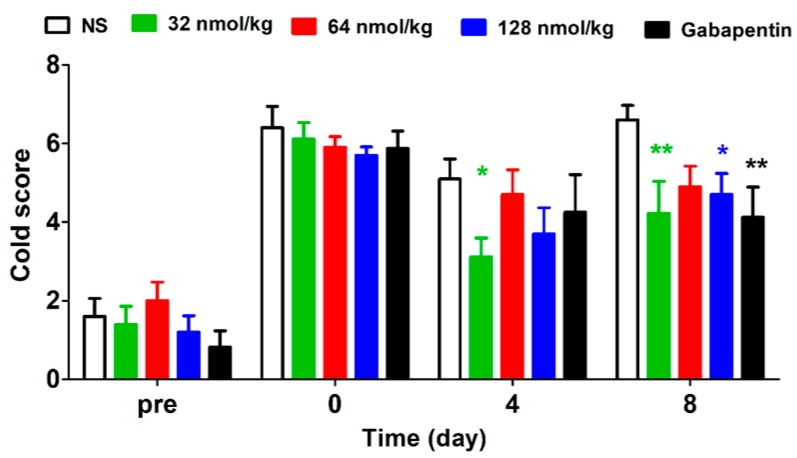
Short-term analgesic effect of GeXIVA[1,2] on oxaliplatin-induced cold allodynia by repeated treatments. Each data point represents the mean ± SEM of cold score at 24 h since last injection (NS, n = 10; GeXIVA[1,2], *n* = 9 (32 nmol/kg), *n* = 10, (64 nmol/kg, 128 nmol/kg); Gabapentin 50 mg/kg, *n* = 8). * *P* < 0.05, ** *P* < 0.01, compared with the NS control group.

Rats from different treatment groups received repeated administration until day 8. The short-term analgesic effects of GeXIVA[1,2] and gabapentin were detected at 24 h post injection. As shown in Figure 6 (F (4,126) = 2.27, *P* = 0.0773), repeated treatment of either GeXIVA[1,2] or gabapentin at 50 mg/kg produced analgesia in cold allodynia. These data suggest a cumulative effect since significant analgesic effects were observed at different doses of GeXIVA[1,2] and gabapentin at day 8 post injection. GeXIVA[1,2] at 32 nmol/kg showed an analgesic effect at day 4 (*P* < 0.05) and day 8 (*P* < 0.01), 128 nmol/kg produced an analgesic effect at day 8 post injection (*P* < 0.05). Gabapentin at 50 mg/kg decreased cold score at day 8 (*P* < 0.01). The cold score of the NS group did not show any significant difference during this period.

#### 2.3.3. Effect of Repeated Treatment of GeXIVA[1,2] in Normal Rats

**Figure 7:**
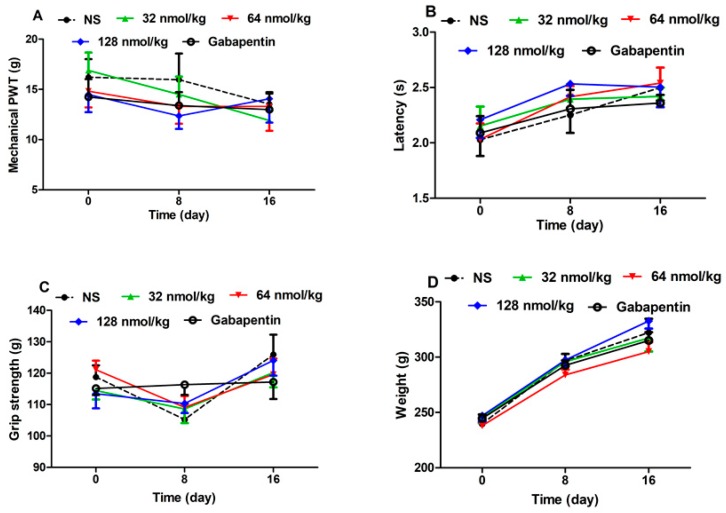
Effect of repeated treatment of GeXIVA[1,2] in normal rats. The changes in mechanical PWT (**A**), tail-flick latency (**B**), grip strength (**C**) and body weight (**D**). Each data point represents the mean ± SEM of pain threshold at 24 h since last injection (NS, *n* = 10; GeXIVA[1,2], *n* = 10 (32 nmol/kg, 64 nmol/kg, 128 nmol/kg); Gabapentin 50 mg/kg, *n* = 10).

In order to evaluate the possible side effects of GeXIVA[1,2] after repeated treatments, mechanical PWT, tail-flick latency, hind limb grip strength and body weight were monitored at day 8 and day 16 in normal rats after daily treatments. As shown in Figure 7, neither a different dose of GeXIVA[1,2] nor gabapentin influenced these indicators after repeated treatments compared with the NS group.

## 3. Discussion

Distinct from neuropathic pain induced by trauma and disease, chemotherapy-induced neuropathic pain is commonly a predictable outcome during cancer treatment due to its high incident rate (68.1% prevalence of neuropathy during the first month after chemotherapy) [21]. Despite the fact that numerous strategies have been investigated for treating this kind of neuropathy, currently only duloxetine is moderately recommended for its treatment [22]. Opioid receptor-targeted drugs are commonly used analgesics to treat acute pain, but they exhibit limitations in chronic neuropathic pain treatment (severe side effects including addiction and respiratory depression, etc.) [23,24].

Peptides derived from toxins target various receptors, such as nAChRs, voltage-gated ion channels, non-opioid G-protein-coupled receptors (GPCRs) and transient receptor potential (TRP) channels. These have been investigated as novel non-opioid analgesic drugs [25]. Ziconotide (ω-conotoxin MVIIA, Prialt®) is a peptide identified from the venom of *conus magus* and approved by the FDA to treat severe chronic pain in 2004. This peptide functions by blocking N-type, voltage-sensitive calcium channels and produces less side effects compared with opioid pain relievers. However, ziconotide could not pass the blood–brain barrier, so it requires an invasive pump for drug delivery to the central nervous system (CNS) which is costly and inconvenient [26].

On the contrary, α9α10 nAChR antagonists modulate neuropathic pain state by peripheral administration, thus they are more convenient than ziconotide. Moreover, unlike opioid therapeutics, α9α10 nAChR antagonists displayed a good safety profile in rodents and clinical tests, which makes this receptor a promising target for chronic neuropathic pain treatment.

Oxaliplatin treatment can induce acute and chronic neuropathic pain. The acute symptoms occur during treatment or within days after treatment, related to impaired functions of calcium sensitive voltage-gated sodium channels. The chronic neuropathic pain symptoms are induced by a cumulative dose of oxaliplatin, related to platinated compound accumulation in dorsal root ganglia-induced neurotoxicity [27]. Previous studies about RgIA and its analogue RgIA4 were performed in the chronic neuropathic pain model; these peptides were repeatedly injected simultaneously with oxaliplatin treatment [16,17]. These results demonstrated the preventive effect of α9α10 nAChR antagonists in oxaliplatin-induced neuropathy. However, considering the cost and necessity, therapeutic treatments are also indispensable. In the present study, we investigated the therapeutic analgesic effect of GeXIVA[1,2] in the established oxaliplatin-induced acute neuropathic pain model.

αO-conotoxin GeXIVA[1,2] is a selective antagonist of α9α10 nAChR with high affinity, and our previous research proposed it as a promising analgesic drug. The present study demonstrated the analgesic efficacy of GeXIVA[1,2] in the oxaliplatin-induced neuropathic pain rat model. We first studied the time/dose–effect relationship of GeXIVA[1,2] in alleviating mechanical and cold allodynia by single IM injection; gabapentin was set as a positive control. GeXIVA[1,2] relieved mechanical allodynia in a dose-dependent manner; 128 nmol/kg GeXIVA[1,2] produced an equivalent analgesic effect to 50 mg/kg gabapentin. On the contrary, GeXIVA[1,2] was inferior in the alleviation of oxaliplatin-induced cold allodynia detected by the acetone test, while 50 mg/kg gabapentin decreased the cold score at 2 h after drug injection. This discrepancy of analgesic efficacy of GeXIVA[1,2] on mechanical and cold allodynia by single injection could be due to multiple reasons. Previous studies were performed as preventive strategies in oxaliplatin-induced chronic neuropathic pain models. In this research, GeXIVA[1,2] was investigated in the established oxaliplatin-induced acute neuropathic pain model as a therapeutic strategy. In an RgIA study, the cold plate test was applied to test cold allodynia, while the acetone test was used in this research [16]. Transient receptor potential ankyrin 1 (TRPA1)-expressing and transient receptor potential melastatin 8 (TRPM8)-expressing sensory fibers mediate mechanical and cold perception respectively [28]. Acute oxaliplatin treatment alters the expression of ion channels in these neurons; however, there is discrepancy in their mechanisms [29].

The analgesic studies of previously identified α9α10 nAChR antagonists place emphasis on their disease-modifying effects, proposing that repeated treatments provide more benefit than single administration [15]. Acute oxaliplatin treatment-induced cold allodynia is reversible, while mechanical allodynia is long-lasting and can continue for at least 20–25 days post treatment [30]. In this study, repeated treatments of GeXIVA[1,2] produce cumulative analgesic effects and promote recovery from oxaliplatin-induced mechanical and cold allodynia. Moreover, the long-term analgesic effect of GeXIVA[1,2] on mechanical allodynia lasted for 10 days after the 16-day repeated treatment procedure was terminated, while gabapentin had no cumulative effects. Pain perception to noxious stimuli is a vital warning signal which should not be affected by pain relievers. GeXIVA[1,2] displayed no effect on normal pain perception detected by the von Frey test and tail flick test after repeated treatment for 16 days, indicating that these analgesic effects are specific to the pathological state.

Although the aforementioned studies suggest that α9α10 nAChR is a promising target to treat neuropathic pain, it is still not known exactly how and where this receptor performs these functions. α9α10 nAChR was initially identified in cochlear hair cells [31,32], and later these subunits’ expression was detected in bone [33], dorsal root ganglion (DRG) [34], liver [35], lymphocytes [36], etc. A recent study identified the expression of α9 and α10 nAChR subunits in mouse brains [37], although this result is controversial [38]. The putative mechanisms involved in the analgesic effect of α9α10 nAChR antagonists are related to the inhibition of immune cell functions [10,12,16]. Several studies have described the immune modulatory effect of α9 containing nAChR. In the experimental autoimmune encephalomyelitis (EAE) model, α9 knockout (KO) mice displayed reduced disease severity and delayed onset [39]. In monocytes, α9 and α10 subunits containing nAChRs regulate purinergic receptor P2 × 7 (P2X7R) activation induced pro-inflammatory cytokine interleukin-1β (IL-1β) release [40]. However, whether these functions are involved in the analgesic effect of GeXIVA[1,2] requires further study.

In the grip strength test, GeXIVA[1,2] did not affect hind limb grip strength after repeated treatments, which is in accordance with our previous data of GeXIVA[1,2] in a rotarod test, indicating that these analgesic effects are not due to neurotoxicity or muscle relaxation [20]. Previously identified α9α10 nAChR antagonists both displayed a good safety profile, and also no significant side effects were observed after single or repeated treatments. RgIA4 had no behavioral, neurological and autonomic side effects in the Irwin test [18]. ZZ1-61c displayed no effect on motor performance and grip strength at the analgesic dose [41]. VC1.1(ACV1) has passed a phase 1 clinical test and been verified as safe for humans [42]. Recently, RgIA4 was licensed to Kineta Inc. (KCP-400) and is in preclinical development [43].

In this study, GeXIVA[1,2] produced an analgesic effect in an oxaliplatin-induced neuropathic pain model. Repeated treatments of GeXIVA[1,2] provided a cumulative effect that can accelerate the recovery from neuropathic pain, which is consistent with previously described α9α10 nAChR antagonists, and is a huge advantage compared with opioid drugs. Compared with ziconotide, GeXIVA[1,2] can be effective by peripheral administration, which is much more convenient and less costly. These results suggest that GeXIVA[1,2] could be a promising drug for chemotherapy-induced neuropathic pain management.

## 4. Material and Methods

### 4.1. Animals

Male Sprague–Dawley rats weighing 180–200 g (Beijing animal center, Beijing, China) were included in the experiments, housed in groups of 5 in transparent plastic cages in a 12 h light/dark cycled, temperature and humidity-controlled room and fed with water and food ad libitum. The animals were given 3–4 days prior to the experiments to adapt to the housing facilities and the testing procedures. All the experiments were approved by the Ethics Committee and the Institutional Animal Care and Use Committee of Beijing Institute of Pharmacology and Toxicology, Beijing, China (IACUC of AMMS-06-2017-001), and were performed according to the ARRIVE (Animal Research: Reporting of In Vivo Experiments) guidelines [44] and Guide for the Care and Use of Laboratory Animals [45] to minimize the quantity of animals used and pain inflicted upon them.

### 4.2. Compounds

Synthesis of αO-conotoxin GeXIVA[1,2] was performed as previously described. Gabapentin (T0702, TargetMol) and oxaliplatin (T0164, TargetMol) were purchased from Target molecule Corp.

### 4.3. Oxaliplatin-Induced Neuropathic Pain

Oxaliplatin (T0164, TargetMol) was dissolved in 5% GS to 2 mg/mL and intraperitoneally (IP) injected at a dose of 6 mg/kg to establish the neuropathic pain model [46]. The control group animals received a corresponding volume of 5% GS injection. Neuropathic pain then gradually emerged as previously described.

### 4.4. Measurement of Mechanical Allodynia

Mechanical allodynia was tested using the von Frey filaments (Aesthesio, Danmic/USA) according to the up–down method as previously described [47,48]. Firstly, all rats were restrained separately in plastic cages placed on a shelf with a metal wire mesh floor and given 15 min for adaptation. A series of von Frey hairs (1–26 g) were applied to the mid-plantar skin of the right hind paw for 2–5 s. A positive response was considered as an urgent, unexpected withdrawal of the paw, then the filament with lower grams was applied; otherwise, the next filament with higher grams was applied. After detecting the first positive and negative response, another 4 pricks were applied. Tests should be avoided when the animals keep moving or show grooming behaviors, which may cause misleading results. The 50% mechanical PWT was calculated by using the formula described previously.

### 4.5. Measurement of Cold Allodynia

Cold allodynia was measured by the acetone test [47,49]. Rats were placed in a transparent plastic box on a shelf with a metal wire mesh floor and given 15 min for habituation. After the rats calmed down and stopped grooming activities, 50 μL acetone was ejected to the mid-plantar skin surface with a syringe, which causes cooling of the skin to a non-nociceptive temperature of 15–21 ℃. The behaviors of rats after the acetone spray was monitored within 30 s and scored according to the following criteria: 0 for no response; 1 for mild paw withdrawal or flicking; 2 for prolonged flicking of hind paw or stomping; 3 for repeated flicking with licking hind paw. This test was repeated 3 times with two-minute intervals and the total scores were recorded.

### 4.6. Measurement of Tail-Flick Latency

The nociceptive perception of heat was detected using a tail-flick analgesia meter (1430, Columbus Instruments, USA). Briefly, the rat was lightly restrained on a platform, and radiant heat was applied to the ventral surface of the tail (4 cm from the distal end of tail); afterwards, the tail-flick latency was recorded automatically. The intensity of heat stimulation was adjusted to obtain a baseline latency of 2 to 5 s; 10 s was set as the cut-off time to avoid tissue damage [20].

### 4.7. Measurement of Grip Strength

In order to evaluate the effect of the test drug on muscle strength, the grip strength test was performed using the grip strength meter (YLS-13A, Yiyan, China). Briefly, the operator grasped the back and the tail of the rat and supported its body against palm, the rat was set at a vertical position and its hind paws were lowered to grasp the grip-bar. After the rat held the grip-bar firmly with both its hind paws, the rat was lowered to a horizontal position and pulled backward slowly. The grip strength was automatically recorded in grams when the rat loosened its grip [50].

### 4.8. Drug Administration Procedure

Based on preliminary results, 32 nmol/kg, 64 nmol/kg and 128 nmol/kg were set as the low, medium, and high dose of GeXIVA[1,2]. To reach these concentrations, GeXIVA[1,2] was dissolved in normal saline to 48 nmol/mL, 96 nmol/mL and 192 nmol/mL and IM injected at a volume of 0.2 mL/300 g body weight. Gabapentin (T0702, TargetMol) was dissolved in NS to 50 mg/mL and IP injected at a volume of 1 mL/kg. On the day of experiment, the animals that developed neuropathic pain symptoms were selected and assigned to different treatment groups randomly. Mechanical or cold sensitivity was monitored at 0, 1, 2, 4, and 6 h after single injection. Afterwards, rats received daily repeated administration for one week, and acetone tests were performed 24 h after the last administrations on day 4 and day 8. In a separate experiment, animals received daily injections of normal saline, GeXIVA[1,2] and gabapentin up to day 16; von Frey tests were performed at 4 h and 24 h since last injection. After drug withdrawal, mechanical PWT was detected for another 16 days. In normal rats, all the test drugs were repeatedly injected for 16 days; mechanical PWT, tail-flick latency, hind paw grip strength and body weight were recorded at day 8 and day 16 before drug injection. The examiner was blinded to drug treatments.

### 4.9. Statistical Analysis

All data were represented as mean ± SEM and analyzed using Graphpad Prism 5. Data including multiple time points were analyzed by two-way ANOVA (time, drug) with Bonferroni post-tests. AUC was calculated by the AUC function of the software, analyzed by one-way ANOVA with Tukey’s multiple comparison. *P* < 0.05 was set as the standard of significance. The acute experiment data represent the pain threshold at 4 h post injection in a repeated treatment process. The short-term effect represents the pain threshold at 24 h since last injection. The long-term analgesic effect represents the pain threshold after drug withdrawal from repeated treatments.
